# Use of mandibular chin bone for alveolar bone grafting in cleft patients

**DOI:** 10.1186/s40902-016-0091-z

**Published:** 2016-11-25

**Authors:** Young-Wook Park, Jang-Ha Lee

**Affiliations:** Department of Oral and Maxillofacial Surgery, College of Dentistry, Gangneung-Wonju National University, 7 Jukheon-Gil, Gangneung, Gangwondo 25457 South Korea

**Keywords:** Alveolar cleft, Alveolar bone grafting, Endochondral bone, Intramembranous bone

## Abstract

**Background:**

We evaluated and compared the outcomes of different ossification processes in patients with alveolar cleft in whom correction was performed using endochondral bone graft or intramembranous bone graft.

**Methods:**

The patients were divided into two groups: the endochondral bone (iliac bone or rib bone) graft group and the intramembranous bone (mandibular bone) graft group. Medical records and radiologic images of patients who underwent alveolar bone grafting due to alveolar cleft were analyzed retrospectively. Through postoperative and follow-up radiologic images, the height of the interdental bone septum was classified into four types based on the highest point of alveolar ridge. Then, the height of the interdental bone septum and the area of the bone graft were evaluated according to the type of bone graft. In addition, the occurrence of complications and the need for an additional bone graft, the result of postoperative orthodontic treatment, and the eruption of impacted teeth were investigated.

**Results:**

Thirty patients were included in this study. There was no significant difference in the change of the interdental bone height and the area of the bone graft according to the type of bone. There was no significant difference in the success rate of the surgery according to the type of bone. One patient underwent an additional bone graft surgery during the follow-up period.

**Conclusions:**

The outcomes of alveolar bone grafting were not significantly different according to the type of bone graft. If appropriate to the size of the recipient site, the chin bone is a useful graft material in alveolar cleft, as is the iliac bone.

## Background

Cleft alveolus is a condition in which there is a break in the continuity of the alveolar process. This condition is usually congenital. Cleft alveolus is the anomaly resulting from the lack of fusion between the medial nasal process and the maxillary process, and it is usually associated with a cleft lip or palate or both [1]. As a result, a problem can occur, such as oral fluid outflow through the nose, nasal secretions entering the mouth, tooth eruption at the rupture site, and alveolar collapse. Cleft alveolus is usually not addressed by the surgical correction of the cleft lip or cleft palate alone. After surgical repair of the cleft lip or cleft palate, the oronasal fistula should be closed and the continuity of the alveolar bone restored. The alveolar bone graft and distraction osteogenesis (DO) are the most common treatments of cleft alveolus [2–4].

DO can reconstruct both the alveolar bone and soft tissue [2, 5]. However, this method increases the treatment period, and DO devices can cause discomfort. Also, additional bone grafting could be necessary in the future. Thus, the alveolar bone graft is still mainly applied for the treatment of cleft alveolus. Through the alveolar bone graft, the aforementioned problems can be solved with intact maxillary arch formation, stabilization of the bone, and the improvement of the face by a proper bone support of the nose and lips [6–9]. The ideal bone graft material for alveolar cleft reconstruction is still controversial. Various bone graft materials such as autogenic, allogenic, xenogenic, and alloplastic grafts have been used in alveolar bone graft. However, autogenic bone is still mainly selected for alveolar bone graft despite the problems of unpredictable atrophy and loss of bone structure [10, 11].

Various types of autogenous bone may be used as grafting materials in alveolar cleft [12]. The iliac bone as the endochondral bone is the most popular, but some authors have reported that the intramembranous bone is more advantageous than the endochondral bone [11, 13, 14]. Hemar et al. performed calvarial bone grafting for maxillofacial reconstruction in 71 patients and had a follow-up of 2 to 6 years [15]. Their results look better than endochondral bone grafting with bones such as the iliac crest, ribs, and tibia. Zins and Whitaker reported that their endochondral bone showed a reduction of three to four times that of intramembranous bone in animal models [16]. It was thought that this difference was caused by the micro-architecture of mineralized matrix and quality of grafted bone. On the other hand, several studies that included long-term observation of cranial bone grafting show no particular advantages compared with iliac bone grafting [17, 18]. As such, there is still controversy regarding the result of alveolar bone graft depending on the type of bone used. Therefore, to get more than a good surgical outcome, you will need to think about the type of bone to be transplanted.

In this retrospective study, we evaluated and compared the outcomes of the different types of ossification processes that were performed using endochondral bone (iliac bone or rib bone) grafting or intramembranous bone (mandibular bone) grafting in alveolar cleft patients. Our goal was to find the most favorable conditions for successful bone grafting.

## Methods

### Patient selection and data collection

This retrospective study was composed of patients who were diagnosed with alveolar cleft and who underwent alveolar bone grafting at the Gangneung-Wonju National University Dental Hospital from January 2007 to December 2013. This study was approved by the Institutional Review Board of the Gangneung-Wonju National University Dental Hospital (IRB 2014-5).

The patients in this study were diagnosed with unilateral or bilateral alveolar cleft and underwent alveolar bone grafting with autogenous bone materials. Patients without 6-month postoperative radiographs were excluded. And patients over the age of 20 years were also excluded from the study. The patients were divided into groups by intramembranous bone graft and endochondral bone graft depending on the ossification of the grafted autogenous bone. The endochondral bone graft was performed from the inlay bone graft into the alveolar cleft site using the corticocancellous block bone, and then the particulate cancellous bone was inserted into the bony gap. The intramembranous bone graft was carried out from the inlay bone graft into the alveolar cleft site using the cortical block bone, and then the crushed cortical bone was filled into the bony gap. Medical and surgical records and radiologic images of patients who were included in this study were analyzed retrospectively. Panoramic and periapical radiographs, preoperative and postoperative radiographs, and follow-up radiographs were compared and evaluated. Postoperative radiographs were taken immediately after surgery, and follow-up radiographs were taken 6 months after surgery. Long-term follow-up radiographs were also taken 1 year after surgery.

### Evaluation of the interdental bone septum height

**Fig. 1:**
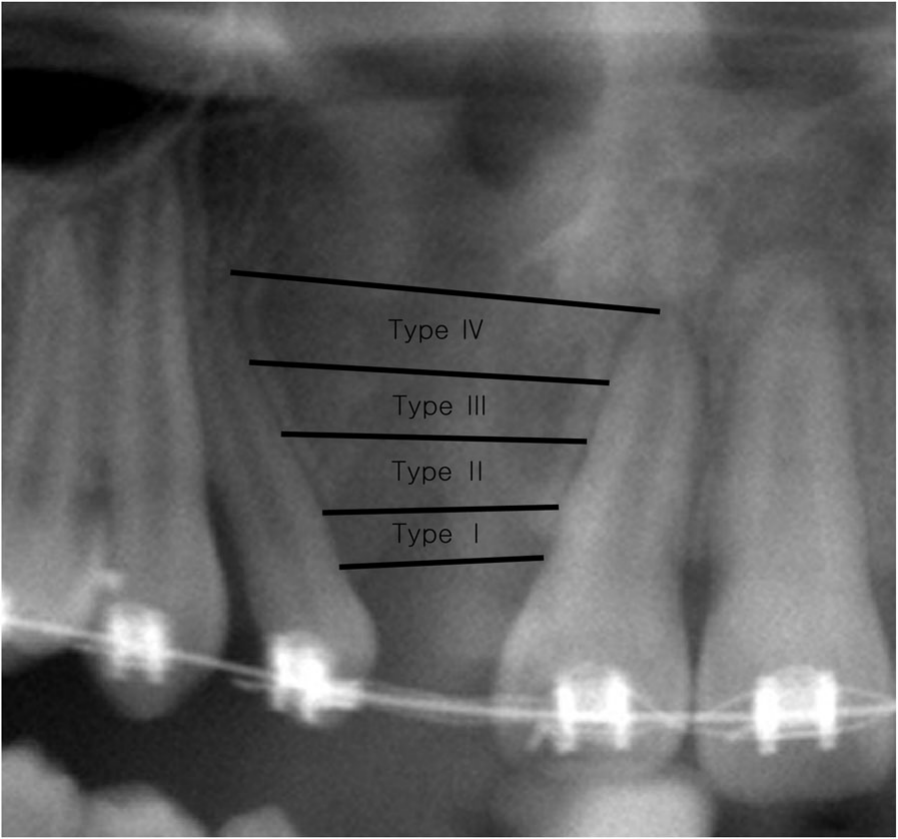
Classification of the type of interdental bone septum between the incisor and canine teeth adjacent to the alveolar cleft site in a preoperative panorama image

Evaluation of the grafting bone was conducted by measuring the inter-alveolar septum height between the incisor and canine teeth adjacent to the cleft via radiographs. The lines between the cervical areas and root apex of the incisor and canine teeth were quartered (Fig. 1). And then, the interdental bone septum height was classified into four types based on the highest point of the interdental bone septum [19, 20]. Type I was more than 75 % of the alveolar ridge height, type II was 50 to 75 % of the alveolar ridge height, type III was less than 50 % of the alveolar ridge height, and type IV has no continuous bony bridge. Each was given a score depending on the type of interdental bone septum. Type I has a score of 4, type II has a score of 3, type III has a score of 2, and type IV has a score of 1. The 6-month follow-up radiographs and the long-term follow-up radiographs were compared, and the differences in the types of grafting bone were evaluated through a comparison of the average score of the interdental bone septum. In evaluating the radiographs 6 months after surgery, the success of the surgery was determined. The criteria of success were determined according to the type of the interdental bone septum: types I and II were evaluated as a success and types III and type IV were determined a failure. In addition, the timing of the alveolar bone grafting was divided by secondary alveolar bone grafting and tertiary alveolar bone grafting according to patient age and a radiograph of each patient, and a success rate was determined.

### Measuring the grafted bone area

**Fig. 2:**
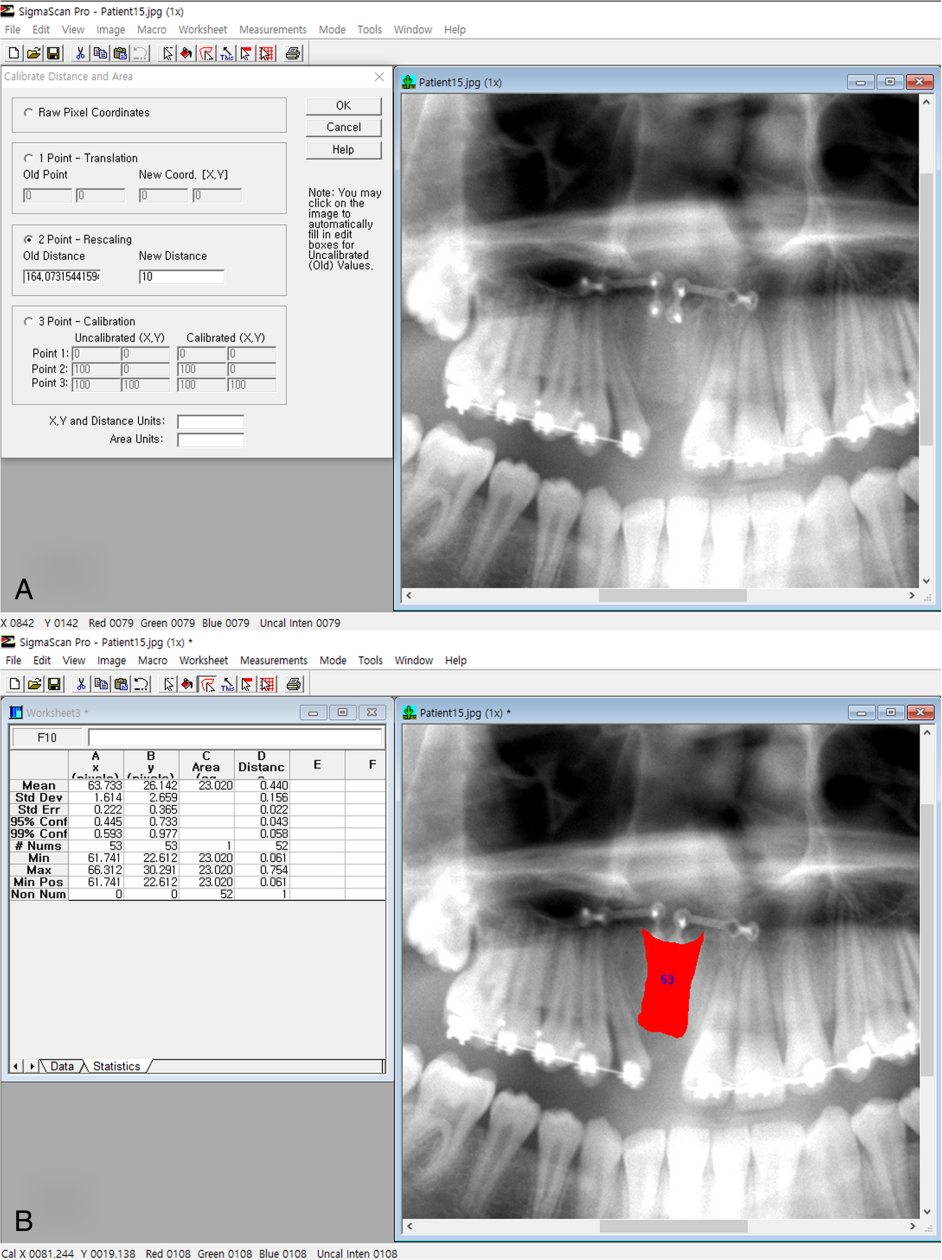
Measuring the area of the graft bone. **a** Calibrating with the long axis of the upper central incisor. **b** Measuring the area of the graft bone

The resorption rate of the graft bone was determined by comparing the area of the bone. The area of graft bone was measured using size-measuring software (SigmaScan-Pro®; SPSS Science, Chicago, IL, USA) in the postoperative radiographs and 6-month follow-up radiographs. After setting the length of the long axis of the upper central incisor as a reference (reference value was 10 mm), the relative area of each bone was measured, and the absorption rate between the postoperative evaluation and 6-month evaluation was calculated (Fig. 2). In addition, the bone surface area that was measured immediately after surgery and 6 months after the surgery was compared. The occurrence of complications, the need for additional bone grafting, and the eruption of the impacted teeth were investigated.

### Statistical analysis

The recorded data were statistically analyzed using IBM SPSS Statistics 23 (IBM Co., NY, USA). The change in the average score of the interdental bone septum over time was analyzed with Mann-Whitney test. And the differences of bone resorption rate were analyzed with independent sample *t* test. The differences between the results of the surgery were analyzed with cross tabulation analysis. The statistical significance level for all tests was considered to be *p* < 0.05.

## Results

Thirty patients were included in this study. Four patients had been excluded by inadequate radiographs, and three patients were excluded because they were over 20 years old. The mean age of patients was 11.27 ± 2.64 years (range, 8–17 years), and 18 patients were female and 12 were male. Seventeen patients underwent the alveolar bone grafting with iliac bone, 12 patients underwent chin bone grafting, and one patient was grafted with the fifth rib bone. The unilateral cleft patients were 27, and the bilateral cleft patients were 3. All bilateral cleft patients were grafted with iliac crest bone (Table 1).

**Table 1:** Classification of cleft, age, gender, and donor site of the patients

Case	Sex	Age	Classification of cleft	Donor site
1	F	9	Unilateral cleft lip and palate	Mandibular symphysis
2	M	9	Unilateral cleft lip and palate	Mandibular symphysis
3	F	10	Unilateral cleft lip	Mandibular symphysis
4	M	10	Unilateral cleft lip	Mandibular symphysis
5	M	11	Unilateral cleft lip and palate	Mandibular symphysis
6	M	11	Unilateral cleft lip and palate	Mandibular symphysis
7	M	11	Unilateral cleft lip and palate	Mandibular symphysis
8	M	11	Unilateral cleft lip and palate	Mandibular symphysis
9	M	12	Unilateral cleft lip and palate	Mandibular symphysis
10	F	12	Unilateral cleft lip and palate	Mandibular symphysis
11	F	14	Unilateral cleft lip	Mandibular symphysis
12	F	15	Unilateral cleft lip and palate	Mandibular symphysis
13	F	8	Unilateral cleft palate	Left ilium
14	M	8	Unilateral cleft lip and palate	Left ilium
15	F	8	Unilateral cleft lip and palate	Left ilium
16	M	9	Unilateral cleft lip and palate	Left ilium
17	M	9	Unilateral cleft lip and palate	Left ilium
18	M	9	Bilateral cleft palate	Left ilium
19	F	9	Unilateral cleft lip and palate	Left ilium
20	F	9	Bilateral cleft lip and palate	Left ilium
21	F	9	Unilateral cleft lip and palate	Left ilium
22	F	11	Bilateral cleft lip and palate	Left ilium
23	F	11	Unilateral cleft lip and palate	Left ilium
24	F	12	Unilateral cleft lip and palate	Left ilium
25	F	13	Unilateral cleft lip and palate	Left ilium
26	F	14	Unilateral cleft lip and palate	Left ilium
27	F	15	Unilateral cleft lip and palate	Left ilium
28	F	16	Unilateral cleft lip and palate	Left ilium
29	F	16	Unilateral cleft lip and palate	Left ilium
30	M	17	Unilateral cleft lip and palate	Right fifth rib

After comparing the height of the interdental bone septum 1 year after surgery, the success rate of the intramembranous bone graft was found to be higher than that of the endochondral bone graft; however, there was no statistically significant difference between the two groups (Table 2). The average interdental bone septum score had no statistically significant difference between the intramembranous bone graft group and endochondral bone graft group postoperatively, at 6-month follow-up and at 1-year follow-up radiographs (Table 3). In addition, even when time had passed, a statistically significant change in the graft bone was not observed in either group. The mean resorption rate of intramembranous bone was higher than that for the endochondral bone, but there was no statistically significant difference in the mean resorption rate between the two groups (Table 4).

**Table 2:** Evaluation of the interdental bone septum and comparison of the success rate after 1 year after surgery according to the ossification type

Type of graft bone	F/U timing after surgery	Type I	Type II	Type III	Type IV	Success rate after 1 year (%)	*χ* ^2^ (*p*)
Intramembranous bone (*n* = 12)	1 week	12	0	0	0	91.67	0.511
	6 months	10	1	1	0		
	1 year	9	2	1	0		
Endochondral bone (*n* = 18)	1 week	16	1	1	0	83.33	
	6 months	15	1	1	1		
	1 year	14	1	1	2		

Types I and II are evaluated as a success (*p* < 0.05). Types III and IV are evaluated as a failure

**Table 3:** Change of mean bone score of the intramembranous bone and endochondral bone over time

Type of graft bone	Mean bone score				
	POD 1W (T1)	POD 6M (T2)	T1–T2 (*p*)^a^	POD 1Y (T3)	T1–T3 (*p*)^a^
Intramembranous bone (*n* = 12)	4.0	3.75 ± 0.62	0.514	3.67 ± 0.65	0.319
Endochondral bone (*n* = 18)	3.83 ± 0.51	3.67 ± 0.84	0.767	3.50 ± 1.04	0.542

Type I has a score of 4, type II has a score of 3, type III has a score of 3, and type IV has a score of 1 (*p* < 0.05)

*POD 1W* evaluation within 1 week after surgery, *POD 6M* evaluation between 3 and 6 months after surgery, *POD 1Y* follow-up evaluation 1 year after surgery

^a^Mann-Whitney test

**Table 4:** Comparison of mean bone resorption rate of the intramembranous bone and endochondral bone at 6 months after surgery

Type of graft bone	Mean resorption rate at 6 months after surgery (%)	*p* value
Intramembranous bone (*n* = 12)	20.71 ± 13.82	NS
Endochondral bone (*n* = 18)	13.23 ± 9.05	

*NS* not significant (*p* < 0.05)

Fifteen patients had received orthodontic treatment: six from the intramembranous bone graft group and nine from the endochondral bone graft group. The space closure was performed by moving the teeth to four of the six patients in the intramembranous bone graft group (66.7 %) and six of the nine patients in the endochondral bone graft group (66.7 %). Nine patients from the endochondral bone graft group had non-erupted teeth, and eight of these patients had non-erupted teeth that erupted after a year. Six patients from the intramembranous bone graft group had non-erupted teeth, and all teeth were erupted after a year. The wound dehiscence occurred in three patients: one from the intramembranous bone graft group and two from the endochondral bone graft group. Although all patients healed during 2 ~ 3 months after surgery; sever reduction of grafted bone occurred until type III or type IV. And one patient received an additional bone graft surgery during the follow-up period. There were no serious complications except for mild infections following surgery in other patients.

## Discussion

In this study, the height of the interdental bone septum in the mesial teeth and distal teeth of the alveolar cleft were compared and evaluated through the radiographs taken immediately after surgery, 6 months after surgery, and 1 year after surgery [11, 19]. This method has been used in several studies to evaluate the results of alveolar bone grafting [21–23]. In this study, if more than 50 % of the graft bones remained 1 year after surgery, we considered it a successful alveolar bone graft. As a result, the success rate of the intramembranous bone graft was 91.67 % and that of the endochondral bone graft was 83.33 %. In comparison with other studies that reported a success rate of 80 to 90 %, both groups showed a similar result [24].

Although not statistically significant, the success rate of intramembranous bone was higher than that of the endochondral bone. Grafted bones were exposed in three patients after surgery. Two patients underwent the iliac crest bone graft, and one patient received the chin bone graft. Severe reduction of the graft bone was observed until type III or type IV in all patients. If the size of the cleft site is large, the excessive tension causes the failure of the primary closure, especially in the palatal side. That is, the size of the alveolar cleft rather than the type of the grafting bone was seen as having a greater effect on the result of the surgery [22, 24].

The interdental bone septum height tended to decrease in the intramembranous bone more than the endochondral bone at 6 months after surgery; however, the endochondral bone decreased more than the intramembranous bone at 1 year after surgery. The mean resorption rate of the area of the grafted bone also tended to decrease in the intramembranous bone more than the endochondral bone at 6 months after surgery. That is, initially, the intramembranous bone is absorbed more rapidly; however, the intramembranous bone is more stable than the endochondral bone in the long-term follow-up. In this regard, one of the most important factors that can affect the outcome of a bone graft is its revascularization. When the graft becomes newly vascularized, nutrients, gas, and undifferentiated mesenchymal cells are transported into the defect and bone regeneration is promoted [25, 26].

In several previous studies, the endochondral bone grafts were more rapidly revascularized than the intramembranous bone grafts in animal models [18, 27]. This would explain the result of the initially greater volume maintenance of the endochondral bone grafts. However, after revascularization, it is considered that that the volume of intramembranous bone is maintained better than that of the endochondral bone due to the differences of micro-architecture of the mineralized matrix of bone [16].

The ilium which can be harvested in large quantities at a time, and is easy to work with due to both the cortical and cancellous bone, is the most popular; however, it has problems such as the gait disturbance and formation of scar tissue around the mouth [28–30]. Some surgeons used the calvarial bone of the intramembranous bone rather than the ilium of the endochondral bone, because of the similarity of the bones’ histology and development [31]. In addition, the autogenous bone harvested from the mandibular ramal or chin area can be used for bone grafting [32]. The mandibular bone has a good result compared to iliac surgery, and it has the advantage of a shorter operative time and hospital stay, as well as no extraoral scar formation [33]. But, if a great amount of grafting bone is required, the mandibular bone cannot be used because only a small amount can be collected. In this study, we used the mandibular bone and not the calvarial bone, and all patients with bilateral cleft received the iliac bone graft.

In this study, the heights of the interdental bone septum were measured 6 months and 1 year after surgery. Other studies have shown that absorption of graft bone occurs mainly during the first 6 months, and there are no significant changes of the bone between 6 months and 1 year following surgery [34]. Therefore, the follow-up period of 1 year is sufficient to test this result. But the limitations of this study were that it has a small number of samples and the width of the bone could not be assessed using radiographic images.

## Conclusions

In this study, the results of the alveolar bone graft were that there is no significant difference according to the type of graft bone used. Although significant bone resorption was observed with the passage of time following alveolar bone grafting, the amount of absorption was not enough to affect the successful outcome. The failure of soft tissue cover in the recipient site largely influences the outcome of the alveolar bone graft. As a result, both the intramembranous bone (mandibular bone) and the endochondral bone (iliac bone or rib bone) can successfully be used for bone grafting of the alveolar cleft. It is important to select the appropriate bone according to the size and shape of the alveolar cleft site and condition of the patient. If appropriate to the size of the recipient site, the chin bone is a useful graft material in the alveolar cleft, as is the iliac bone.
